# Prophylactic Mesh Reinforcement Versus Primary Suture for Midline Laparotomy Closure: An Updated Systematic Review and Meta-Analysis

**DOI:** 10.7759/cureus.104109

**Published:** 2026-02-23

**Authors:** Marwan Ibrahim, Ali Alseneid, Mara Mijatovic, Ahmed Sultan

**Affiliations:** 1 Department of General Surgery, Milton Keynes University Hospital, Milton Keynes, GBR; 2 Department of General Surgery, William Harvey Hospital, Ashford, GBR; 3 Faculty of Medicine, University of Montpellier, Montpellier, FRA

**Keywords:** abdominal wall closure, elective laparotomy, incisional hernia prevention, incisional hernia repair, meta-analysis, midline laparotomy, prophylactic mesh, systematic review

## Abstract

Incisional hernia remains a common postoperative complication following elective midline laparotomy. Prophylactic mesh reinforcement has been proposed to reduce hernia formation, although concerns regarding postoperative complications and variation in operative technique have limited routine adoption. This systematic review and meta-analysis of randomized controlled trials (RCTs) was conducted according to Preferred Reporting Items for Systematic Reviews and Meta-Analyses (PRISMA) 2020 guidelines and registered on PROSPERO (CRD420251172749). A comprehensive search identified RCTs comparing prophylactic mesh reinforcement with primary suture closure in elective midline laparotomy. The primary outcome was incisional hernia incidence at ≥12 months follow-up. Data were pooled using fixed-effects meta-analysis. Four RCTs comprising 1,006 patients met criteria for quantitative synthesis. Prophylactic mesh significantly reduced the incidence of incisional hernia compared with primary suture closure (relative risk (RR) 0.39, 95% confidence interval (CI) 0.27-0.57). Heterogeneity was moderate (I² = 44.6%). Observational studies supported similar trends without evidence of increased clinically significant wound morbidity. Prophylactic mesh reinforcement reduces the risk of incisional hernia following elective midline laparotomy without clear evidence of increased postoperative complications. Selective use in high-risk patients may improve long-term abdominal wall outcomes, although further standardized studies are warranted.

## Introduction and background

Incisional hernia is among the most frequent long-term complications following elective midline laparotomy, with reported rates ranging from 10% to 20% depending on patient comorbidities, closure technique, and duration of follow-up [1,2]. These hernias can lead to discomfort, functional impairment, cosmetic concerns, and the potential need for complex abdominal wall reconstruction, placing a substantial burden on both patients and healthcare systems [3]. As surgical case complexity and patient risk profiles evolve, preventing incisional hernia has become an increasingly important focus in perioperative surgical care and long-term patient outcomes.

Prophylactic mesh reinforcement has been proposed as a strategy to reduce the risk of fascial failure by augmenting the tensile strength of the abdominal wall closure [1,2]. Several randomized and observational studies have suggested that mesh reinforcement may lower the incidence of incisional hernia, particularly in high-risk patients or in settings where wound tension is elevated [4,5]. However, adoption has been cautious due to concerns about mesh-related complications, including infection, seroma formation, and chronic pain. Variation in mesh materials, placement techniques, and patient selection criteria has further contributed to inconsistent practice patterns across institutions and surgical specialties.

Previous systematic reviews evaluating prophylactic mesh have often included heterogeneous study populations, combining elective and emergency surgery, contaminated fields, and non-midline abdominal incisions [6-9]. Such variability limits the ability to draw firm conclusions for routine elective midline laparotomy, in which surgical exposure, wound environment, and postoperative risks differ significantly from emergency procedures. Additionally, more recent randomized trials with longer follow-up and improved methodology have provided new insights that were not incorporated into earlier reviews.

Given these developments, an updated synthesis focused specifically on elective midline laparotomy is warranted. The aim of this systematic review and meta-analysis was to evaluate the effectiveness of prophylactic mesh reinforcement compared with primary suture closure in preventing incisional hernia after elective midline laparotomy, using the highest-quality evidence available and incorporating contemporary trial data to provide a clinically focused and methodologically rigorous assessment of this preventive strategy.

## Review

Methodology

Protocol and Registration

This systematic review and meta-analysis review was conducted in accordance with Preferred Reporting Items for Systematic Reviews and Meta-Analyses (PRISMA) 2020 guidelines [10] and was prospectively registered with PROSPERO (CRD420251172749) [11]. Adult patients undergoing elective midline laparotomy were included. The intervention of interest was prophylactic mesh reinforcement compared with primary suture closure. Randomized controlled trials (RCTs) were eligible for quantitative synthesis. Observational studies were included solely to provide contextual safety and feasibility data and were not incorporated into the pooled meta-analysis.

Search Strategy

Searches were conducted in PubMed, Embase, Scopus, and the Cochrane Central Register of Controlled Trials using predefined keywords relating to prophylactic mesh and incisional hernia. Only English-language studies were included due to feasibility and resource constraints related to translation. Studies involving emergency surgery, contaminated operations, non-midline incisions, pediatric patients, or insufficient outcome data were excluded.

Study Selection and Data Extraction

Two reviewers (MI and AA) independently screened all titles and abstracts, followed by full-text review, with discrepancies resolved through discussion and consensus. Data extraction included study characteristics, operative technique, mesh type and position, comparator details, follow-up duration, and hernia outcomes. The primary outcome was incisional hernia occurring at a minimum follow-up of twelve months. Secondary outcomes were synthesized narratively due to inconsistent definitions and reporting across studies.

Risk-of-Bias Assessment

Risk of bias for randomized trials was assessed using the Cochrane RoB 2 tool. This tool evaluates bias across several domains, including the randomization process, deviations from intended interventions, missing outcome data, outcome measurement, and selective reporting. Any uncertainties in the assessment were resolved through discussion among the authors.

Certainty of Evidence Assessment

Certainty of evidence for the primary outcome was assessed using the GRADE approach. This framework considers multiple factors, including risk of bias, inconsistency, indirectness, imprecision, and potential publication bias, to determine the overall strength and reliability of the evidence.

Statistical Analysis

A fixed-effects model was used to pool risk ratios with 95% confidence intervals. Statistical heterogeneity was evaluated using the I² statistic, with values above 50% considered indicative of substantial heterogeneity. Secondary outcomes were synthesized narratively, where inconsistent definitions or reporting precluded quantitative meta-analysis.

Results

Study Selection

**Figure 1:**
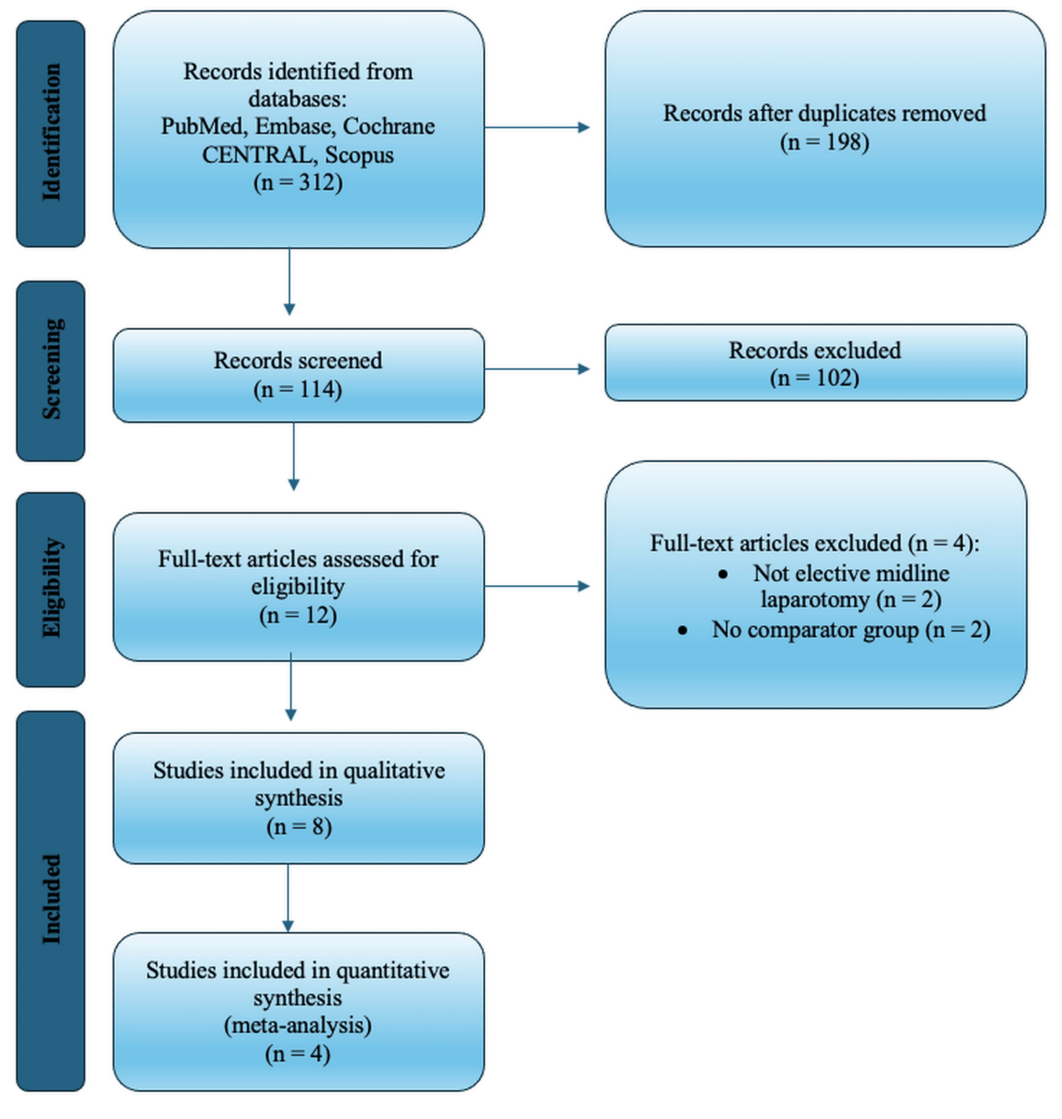
Preferred Reporting Items for Systematic Reviews and Meta-Analyses (PRISMA) 2020 flow diagram Summarizing the identification, screening, eligibility assessment, and final inclusion of studies in the systematic review.

A total of 312 records were identified through database searches, of which 198 remained after removal of duplicates. Following title and abstract screening, 12 full-text articles were reviewed, and eight met the criteria for inclusion. Four RCTs were eligible for quantitative synthesis, and five observational studies were included in the qualitative analysis (Figure 1). 

Characteristics of RCTs

The randomized trials enrolled a combined total of 1,006 patients undergoing elective midline laparotomy and compared prophylactic mesh reinforcement with primary suture closure. These trials differed in mesh type and position, with studies evaluating onlay mesh reinforcement, sublay reinforcement, and intraperitoneal onlay mesh. Follow-up durations ranged from twelve months to five years. Detailed characteristics of the randomized trials are presented in Table 1.

**Table 1:** Characteristics of the randomized controlled trials included in the quantitative synthesis The table is detailing study design, sample size, mesh type and position, comparator technique, and follow-up duration. AAA: abdominal aortic aneurysm, BMI: body mass index

Study	Population	Sample size (mesh / suture)	Intervention (mesh type and position)	Comparator	Follow-up	Primary outcome
Bevis et al., 2010 [12]	Elective open AAA repairs (midline laparotomy)	Mesh: 37 Suture: 43	Retrorectus (sublay) polypropylene mesh reinforcement	Primary suture closure	24 months	Incisional hernia
Caro-Tarragó et al., 2014 [4]	Elective midline laparotomy for general abdominal surgery	Mesh: 80 Suture: 80	Onlay polypropylene mesh reinforcement	Primary continuous suture closure	12 months	Incisional hernia
Jairam et al., 2017 [5]	Elective midline laparotomy in high-risk patients (BMI ≥27 kg/m² or AAA)	Mesh: 188 Suture: 107 (onlay vs suture)	Large-pore polypropylene onlay prophylactic mesh	Primary suture closure	24 months	Incisional hernia
Glauser et al., 2019 [13]	Mixed elective abdominal surgery	Mesh: 95 Suture: 88	Intraperitoneal onlay mesh (IPOM) “mesh-strip” technique	Primary suture closure	5.3 years	Incisional hernia

Quantitative Synthesis (Meta-Analysis)

**Figure 2:**
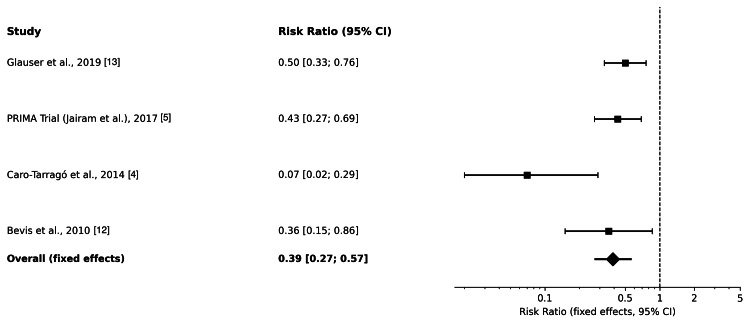
Forest plot comparing prophylactic mesh reinforcement with primary suture closure for the prevention of incisional hernia following elective midline laparotomy Individual study risk ratios with 95% confidence intervals are shown as squares and horizontal lines, respectively. The pooled effect estimate is displayed as a diamond. A fixed-effects model was used, and the vertical dashed line represents the line of no effect (risk ratio = 1).

Meta-analysis of the four randomized trials demonstrated that prophylactic mesh reinforcement significantly reduced the incidence of incisional hernia compared with primary suture closure (relative risk (RR) 0.39, 95% confidence interval (CI) 0.27-0.57). Heterogeneity across studies was moderate (I² = 44.6%). Hernia reduction was consistent across mesh types and positions, and no individual trial showed a reversal of the overall direction of effect. The pooled results are shown in Figure 2.

Characteristics of Observational Studies

Three observational studies were included to provide contextual evidence regarding the safety and effectiveness of prophylactic mesh reinforcement following laparotomy. These studies encompassed emergency and high-risk abdominal surgery populations and varied in design, follow-up duration, and mesh positioning techniques.

Alsaadi et al. [14] reported a retrospective single-surgeon cohort of 24 patients undergoing emergency laparotomy with prophylactic onlay mesh reinforcement as part of a standardized abdominal wall closure bundle. Early postoperative outcomes demonstrated low wound morbidity, with surgical site infection and seroma each occurring in approximately 4% of patients. These findings suggest that prophylactic mesh placement may be feasible in emergency settings without substantial short-term complication burden.

Bravo-Salva et al. [15] conducted a retrospective comparative observational study including 187 patients undergoing emergency midline laparotomy. Patients receiving prophylactic mesh reinforcement demonstrated a significantly lower incidence of incisional hernia compared with primary suture closure (approximately 14% versus 37%) at long-term follow-up exceeding five years. Chronic mesh-related complications were uncommon, and no mesh explantation was required.

Rhemtulla et al. [16] performed a matched retrospective cohort study evaluating prophylactic mesh augmentation in high-risk laparotomy patients (18 mesh vs. 75 matched controls). Short-term incisional hernia incidence was lower in the mesh group (0% vs approximately 5%), with no significant increase in surgical site infection or other wound complications. These findings further support the potential safety and benefit of prophylactic mesh reinforcement in selected high-risk patients. Characteristics of these observational studies are summarized in Table 2.

**Table 2:** Characteristics of the observational studies included in the qualitative synthesis The table is summarizing study design, patient population, mesh technique, follow-up duration, and key reported outcomes. Comparator refers to patients undergoing primary suture closure without prophylactic mesh reinforcement unless otherwise specified. SSI: surgical site infection, TIGR®: long-acting synthetic resorbable mesh

Study	Design and setting	Sample size	Mesh type and position	Comparator	Follow-up duration	Key outcomes
Alsaadi et al. [14]	Retrospective single-surgeon cohort; emergency laparotomy	24 patients	Long-acting synthetic resorbable mesh (TIGR®), onlay placement	None (single-arm cohort)	Early postoperative follow-up (~weeks to months)	SSI ~4%; seroma ~4%; overall complication rate low, suggesting acceptable short-term safety in emergency settings
Bravo-Salva et al. [15]	Retrospective comparative observational study; emergency midline laparotomy	187 patients (mesh 56, suture 131)	Synthetic lightweight partially absorbable mesh, onlay position	Primary suture closure	Median ~64 months	Incisional hernia ~14% mesh vs ~37% suture; minimal chronic mesh complications; no mesh explantation required
Rhemtulla et al. [16]	Retrospective matched cohort study; high-risk laparotomy patients	18 mesh vs 75 matched controls	Synthetic prophylactic mesh augmentation (onlay reinforcement)	Primary closure controls	Mean ~6 months	Hernia incidence 0% mesh vs ~5% controls; SSI ~11% mesh vs ~19% controls; no significant increase in wound complications

Risk-of-Bias Assessment

**Figure 3:**
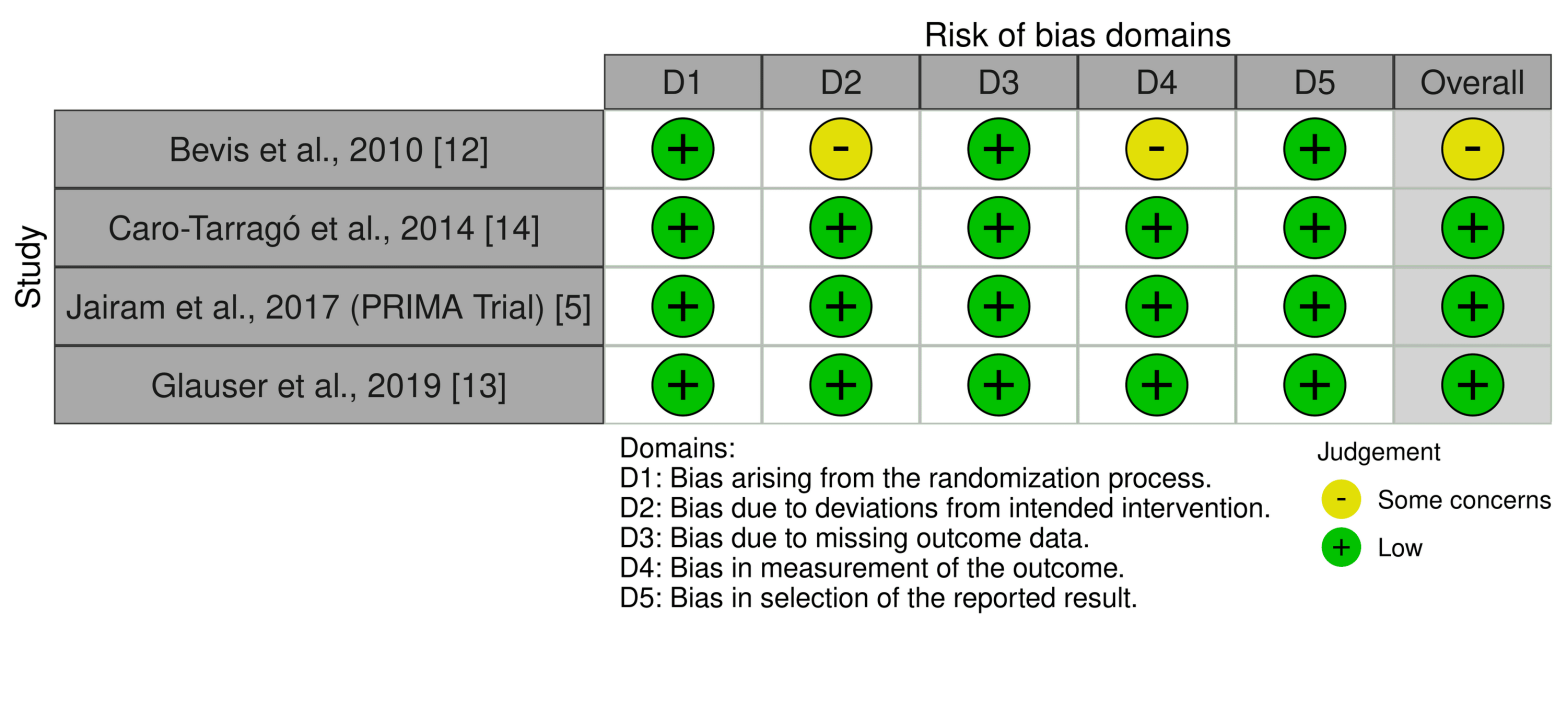
Risk-of-bias traffic light plot for the included randomized controlled trials The plot is illustrating domain-level judgments using the Cochrane RoB 2 tool displaying the proportion of studies rated as low risk, some concerns, or high risk for each bias domain.

Risk-of-bias analysis identified three trials at low risk of bias across most domains and one trial with some concerns related primarily to outcome assessment and completeness of follow-up. Overall, the included randomized trials demonstrated generally robust methodological quality, with appropriate randomization procedures and minimal missing outcome data. Graphical summaries of risk-of-bias judgments are presented in Figure 3, with detailed domain-level assessments provided in Table 3.

**Table 3:** Risk-of-bias assessment of the randomized controlled trials using the Cochrane RoB 2 tool, with judgments for each methodological domain

Study (year)	Bias arising from randomization	Bias due to deviations from intended interventions	Bias due to missing outcome data	Bias in the measurement of the outcome	Bias in the selection of the reported result	Overall risk of bias
Bevis et al., 2010 [12]	Low	Some concerns (open-label surgical trial)	Low	Some concerns (clinical assessment without blinding)	Low	Some concerns
Caro-Tarragó et al., 2014 [14]	Low	Low	Low	Low	Low	Low
PRIMA Trial (Jairam et al., 2017) [5]	Low	Low	Low	Low	Low	Low
Glauser et al., 2019 [13]	Low	Low	Low	Low	Low	Low

Secondary Outcomes

Secondary outcomes were reported inconsistently among the randomized trials [12,13]. Rates of surgical site infection did not differ significantly between mesh and suture groups in the trials that reported this outcome [5]. Seroma formation was more frequently observed in some mesh groups, although reporting definitions varied, and most events were classified as minor or self-limiting [4,5]. Early postoperative complications such as hematoma, wound dehiscence, or reoperation were uncommon and showed no consistent differences between groups [5,12]. Observational evidence supported these findings, with no clear increase in clinically significant morbidity associated with mesh use. 

Certainty of Evidence

The overall certainty of evidence for the primary outcome was graded as moderate using the GRADE framework, primarily due to moderate heterogeneity across included trials. There were no major concerns regarding risk of bias, indirectness, or imprecision for this outcome. Certainty for secondary outcomes was lower due to inconsistent reporting, variability in outcome definitions, and limited comparability across studies. The detailed certainty assessments are summarized in Table 4.

**Table 4:** GRADE summary of findings for the primary outcome of incisional hernia at a minimum follow-up of 12 months, including effect estimates, certainty ratings, and justification for grading decisions RCT: randomized controlled trials, RR: relative risk, MD: mean difference

Outcome	Effect (RR or MD)	Absolute effect	No. of participants (studies)	Certainty of evidence (GRADE)	Justification
Incisional hernia at ≥12 months	RR 0.39 (95% CI 0.27–0.57)	Mesh reduces hernia from 20% to ~8%	1,006 participants (4 RCTs)	Moderate certainty	Downgraded one level for heterogeneity (I² = 45%). No serious concerns for risk of bias, indirectness, or imprecision.
Surgical site infection (SSI)	Not meta-analyzed	Inconsistent definitions and reporting	4 RCTs (Inconsistently reported)	Low certainty	Downgraded for inconsistency and indirectness. No pooled analysis possible due to outcome heterogeneity.
Seroma formation	Not meta-analyzed	Variable definitions; inconsistent follow-up	4 RCTs	Low certainty	Downgraded for large variation in reporting, detection bias, and imprecision.
Early postoperative complications (composite)	Narrative only	No consistent effect across studies	4 RCTs	Low certainty	Downgraded for outcome heterogeneity, unclear measurement, and selective reporting differences.

Discussion

This systematic review and meta-analysis provide updated evidence showing that prophylactic mesh reinforcement significantly reduces the incidence of incisional hernia after elective midline laparotomy. Across the included RCTs, the direction and magnitude of the treatment effect were consistent, and the pooled estimate demonstrates a substantial reduction in hernia formation. These findings reinforce the concept that midline closure benefits from mechanical reinforcement in selected patients, particularly those with risk factors for fascial failure.

Previous reviews have reported similar benefits of prophylactic mesh, but many combined heterogeneous surgical populations, including emergency procedures and non-midline incisions, which limits their applicability to purely elective midline surgery [6,7,9]. By focusing exclusively on elective midline laparotomy and incorporating more recent randomized data, the present review offers a more targeted and contemporary assessment of this strategy. The observed effect size suggests that prophylactic mesh can substantially decrease the long-term burden of incisional hernia when used in appropriate patients. This is consistent with other recent systematic reviews demonstrating significant reductions in incisional hernia rates across multiple follow-up intervals, including one-, two-, three-, and five-year outcomes [17].

Concerns regarding mesh-associated complications have historically contributed to hesitancy in adopting prophylactic reinforcement. In this review, clinically important wound problems were not consistently increased among mesh recipients [12,13]. Although minor seroma formation was more frequent in some reports, these events were generally self-limiting and did not appear to affect long-term outcomes [4, 5, 8]. Observational data included in the qualitative synthesis, together with larger prospective cohorts evaluating mesh in abdominal wall surgery, support an acceptable safety profile when mesh is used in carefully selected patients and within standardized perioperative pathways [14-16]. Broader meta-analytic evidence also suggests that while prophylactic mesh may increase seroma formation or chronic wound discomfort in some settings, overall benefits in hernia prevention tend to outweigh these risks in appropriately selected patients [17].

Limitations

Despite these strengths, important uncertainties remain. The included studies differed in mesh materials, positioning techniques, and criteria for patient selection, which makes it difficult to define a single optimal approach for all patients. Follow-up duration varied, and late hernia development may not have been fully captured in every trial. In addition, secondary outcomes such as surgical site infection, seroma, and broader postoperative complications were reported inconsistently, limiting detailed comparative analysis. The incorporation of observational studies, while useful for contextual safety assessment, introduces potential confounding and selection bias. Future research should prioritize harmonized outcome definitions, longer-term follow-up, and head-to-head comparisons of mesh position and material. There is also a need to establish validated risk stratification tools to guide which patients stand to benefit most from prophylactic reinforcement.

## Conclusions

Prophylactic mesh reinforcement demonstrates a clear and clinically meaningful reduction in the incidence of incisional hernia following elective midline laparotomy. The consistency of effect across randomized trials supports the reliability of this intervention as a strategy to strengthen midline fascial closure and reduce long-term structural failure. Importantly, the available evidence does not show a substantial increase in clinically significant postoperative complications, suggesting that prophylactic mesh appears safe when applied in appropriate patient populations and under controlled operative conditions.

The findings of this review align with broader systematic evidence demonstrating durable reductions in incisional hernia rates across multiple follow-up periods, reinforcing the potential value of prophylactic reinforcement in high-risk surgical populations. Observational data further suggest that this approach can be integrated into real-world practice when careful patient selection and standardized perioperative protocols are used.

Despite encouraging results, further research is needed to define optimal mesh type, placement technique, and patient selection criteria. Longer follow-up studies, standardized outcome reporting, and evaluation of patient-centered outcomes such as quality of life and functional recovery will be essential. Overall, this review supports the selective use of prophylactic mesh reinforcement in elective midline laparotomy while highlighting the need for continued refinement of surgical technique and evidence-based implementation strategies.
